# Benefits of Antimicrobial Photodynamic Therapy as an Adjunct to Non-Surgical Periodontal Treatment in Smokers with Periodontitis: A Systematic Review and Meta-Analysis

**DOI:** 10.3390/medicina59040684

**Published:** 2023-03-30

**Authors:** Kelly R. V. Villafuerte, Cristhiam Jesus H. Martinez, Luiz H. Palucci Vieira, Atila V. Nobre

**Affiliations:** 1 Centro de Investigación en Biodiversidad para la Salud—Biocentro, de la Universidad Privada Norbert Wiener, Lima 15108, Peru; 2 Department of Oral and Maxillofacial Surgery and Periodontology, School of Dentistry, University of São Paulo, Ribeirão Preto 14040-904, Brazil; 3 Department of Physical Education, Faculty of Sciences, São Paulo State University, Bauru 17033-360, Brazil; 4 RENACYT Independent Researcher, Lima 15086, Peru

**Keywords:** photodynamic therapy, ּscaling root planning, smokers, periodontitis

## Abstract

The objective of this study was to analyze evidence of the clinical and microbiological benefits of antimicrobial photodynamic therapy (aPDT) adjunctive to scaling and root planing (SRP) in smokers with periodontitis. Randomized clinical trials (RCTs) were included, through an electronic search in PubMed/MEDLINE, LILACS, Web of Science, and the Cochrane Library for articles published in English until December 2022. The quality of the studies was assessed using the JADAD scale and the risk of bias was estimated using the Cochrane Collaboration assessment tool. Of the 175 relevant articles, eight RCTs were included. Of these, seven reported clinical results and five microbiological results, with a follow-up time of 3–6 months. A meta-analysis was performed for the probing depth (PD) reduction and clinical attachment level (CAL) gain at 3 and 6 months. The weighted mean differences (WMDs) and 95% confidence intervals (CIs) were counted for the PD and CAL. The overall effect for the PD reduction at 3 and 6 months (WMD = −0.80, 95% CI = −1.44 to −0.17, *p* = 0.01; WMD = −1.35, 95% CI = −2.23 to −0.46, *p* = 0.003) was in favor of aPDT. The CAL gain (WMD = 0.79, 95% CI = −1.24 to −0.35, *p* = 0.0005) was statistically significant at 6 months, in favor of aPDT. In these RCTs, aPDT was unable to demonstrate efficacy in reducing the microbial species associated with periodontitis. aPDT as an adjuvant to SRP improves the PD reduction and CAL gain more effectively than only SRP. RCTs are needed to establish standardized protocols with longer follow-up times in order to provide more results on aPDT adjunctive to SRP in smokers with periodontitis.

## 1. Introduction

The oral cavity is colonized by hundreds of microbial species, grouped into complex communities that live in homeostasis with the host [1]. However, these microbial communities can suffer changes caused by poor dental hygiene and the combination of various local and systemic factors related to the host [2]. Among them, smoking is one of the main local risk factors that favor the development and progression of periodontitis [3,4].

Periodontitis is a chronic inflammatory disease that affects the tooth’s supporting tissues [5,6]. The development of this disease is accompanied by profound changes in the composition of the biofilm [7]. A change in this balance is quantitative and qualitative as a result of the competitiveness between species, leading to an increase in the proportion of pathogenic bacteria [5] and causing dysbiosis and host immune/inflammatory responses that exacerbate periodontal destruction [4,5,7].

In recent years, a comparison of genetic and epigenetic polymorphisms in periodontal disease between smokers and non-smokers suggested an increased risk of disease for tobacco users [8]. Furthermore, there is evidence in the literature that smokers have a higher rate of attachment loss and bone loss [9,10], a higher number of deep periodontal pockets, and a higher number of pathogenic microorganisms [11], due to immunosuppression and/or nicotine decreasing the local oxygen tension and favoring the multiplication of periodontal pathogens [9,11].

The standard therapy for periodontitis is the non-surgical removal of the biofilm by scaling and root planing (SRP), which usually leads to clinical improvement and a healthy microbiota [12]; however, the effectiveness of SRP can be compromised in areas of deep pockets and complex root anatomy such as a furcation lesion; therefore, adjuvant approaches are needed [13]. Thus, photodynamic antimicrobial therapy (aPDT) emerges as a method of microbial reduction and is beneficial in areas that are difficult to access, such as deep periodontal pockets or furcation lesions, and it is unlikely that a microorganism will develop bacterial resistance; thus, showing it as an adjuvant alternative to periodontal treatment [14].

The treatment of aPDT is characterized by the combination of a light source and a photosensitizer, which, after absorbing the light energy and initiating chemical reactions, produces free oxygen radicals (singlet), producing a highly reactive toxic effect, resulting in cellular necrosis and death by oxidative stress [15]. PDT has been used in different medical areas to treat a variety of conditions, including cancer [16]. In the case of periodontal disease, aPDT is used as an adjuvant treatment to traditional scaling and root planing. Studies have found that aPDT not only has a bactericidal effect, but also an anti-inflammatory effect on the periodontal tissues, by reducing the number of inflammatory mediators to provide a more favorable healing environment, as well as restoring the cellular biological balance [14,17].

Additionally, studies have demonstrated that aPDT improves the effect of periodontal treatment, by reducing periodontopathogenic microorganisms, due to its potent bactericidal activity [14,18,19,20]. In this way, clinical studies have emerged in patients with periodontitis who smoke to observe the benefit of aPDT within clinical, immunological, and microbiological parameters. However, authors have reported conflicting results, as some show a significant improvement in terms of clinical and microbiological parameters [20], while others provide only modest clinical improvement without significant microbiological changes [21].

In this context, the objective of this study was to analyze the evidence of the clinical and microbiological benefits of antimicrobial photodynamic therapy (aPDT) as an adjunct to SRP in smokers with periodontitis.

## 2. Methods

This systematic review was structured according to the Preferred Items for Systematic Reviews and Meta-Analyses (PRISMA) guidelines and registered with the National Institute of Health Research PROSPERO, International Prospective Register of Systematic Reviews (registration CRD42020183466, available on 5 July 2020 https://www.crd.york.ac.uk/prospero/display_record.php?RecordID=183466).

### 2.1. Inclusion Criteria

Types of studies to be included: Randomized clinical trials (RCTs), published in English, were considered eligible for inclusion.

(P)articipants/population: Adult smokers diagnosed with periodontitis.

(I)nterventions/exposure: The use of antimicrobial photodynamic therapy (aPDT) as an adjuvant to non-surgical periodontal therapy (scaling and root planning—SRP + aPDT).

(C)omparator(s)/control: Only non-surgical periodontal therapy (SRP).

(O)utcome measures:

Primary outcomes:

- Changes in periodontal clinical parameters (reduction in pocket depth (PD) and gain in clinical attachment level (CAL)).
- Changes in microbiological parameters (changes in the bacterial population—bacterial count).

Effect measures:

- PD and CAL in millimeters (mm).
- Changes in microbiological proportion and/or percentage.

### 2.2. Secondary Outcomes

Percentage changes in bleeding on probing (BOP) and the plaque index (PI) as secondary outcomes.

Exclusion criteria: (1) Studies with other adjunctive therapies besides antimicrobial photodynamic therapy, such as the local or systemic administration of antibiotics; (2) in vitro (laboratory) studies and animal models; (3) studies that include patients with any other type of systemic disease or autoimmune diseases (for example, diabetes, cardiovascular disease, and rheumatoid arthritis); (5) studies that include pregnant patients; (6) studies with any antimicrobial solutions; (7) case reports, letters, and reviews.

Research strategy: The research included all articles indexed in PubMed/MEDLINE, LILACS, Web of Science, and the Cochrane Library published in the English language. The electronic search was conducted on 1 May 2020 and updated on 24 December 2022, using different combinations of the following descriptors and/or Medical Subject Headings (MeSH), “photodynamic therapy” and “laser”, each combined with the Boolean operators (OR, AND): “periodontal diseases” OR “periodontitis” OR “periodontitis adult” AND “scaling root planing” OR “periodontal debridement” OR “root planning” OR “non-surgical periodontal therapy” OR “periodontal treatment” AND “cigarette smoking” OR “smoking” OR “smoking, cigarette” OR “tobacco” OR “smoking, tobacco”.

After the electronic search, manual searches were conducted in the reference lists of the selected articles.

Study selection: The study selection and data extraction process was in accordance with the PRISMA guidelines. The titles and abstracts of the identified studies were selected by two reviewers (KRVV and CHM). Disagreements between the reviewers were resolved through discussion or consultation with a third reviewer (AVN). Studies with insufficient information in the title and abstract were selected for evaluation of the full report, which was performed independently by the same two reviewers to determine the eligibility of the studies. In the absence of data, if necessary, the authors were contacted through email.

### 2.3. Extracted Data

The relevant data extracted from all the studies included: author name(s) and publication year; country; study design; sample size; types and concentration of photosensitizer; photosensitizer time; type of laser; laser parameters and settings; administration of PDT (time and the number of applications); participant characteristics; type of periodontitis and definition; interventions and follow-up; criteria for smoking/years of smoking; periodontal parameters; sample location; microbiological techniques; collection time; follow-up, evaluated bacteria; measures of the outcome of interest; and source of funding.

To obtain data that was missing in the reports, if necessary, the authors of the included studies were contacted. The GetData Graph Digitizer 2.26 was also used (http://getdata-graph-digitizer.com, (software downloaded on 23 December 2022). to read the data that was only illustrated in figures [22].

### 2.4. Risk of Bias Assessment

The risk of bias in the included studies was evaluated according to the Cochrane Collaboration’s Risk of Bias tool. The methodological quality of the included studies was assessed according to the JADAD score [23] and was performed by two authors (KRVV and CHM) and in which the studies were classified with scores ranging from 0 to 5. A score of 3 or higher equated to high methodological quality, studies with a score of 2 or less were considered of low methodological quality.

The risks of bias were classified as adequate (+), inadequate (−), or unclear (?). The methods of randomization and allocation (selection bias); patient blinding (performance bias); operators and examiners (detection bias); completeness of follow-up period/incomplete outcome data (attrition bias); selective reporting (reporting bias); and others were assessed based on these domains. The overall risk of bias was categorized as follows: (1) low risk of bias if all criteria were met; (2) unclear risk of bias if one or more criteria were partially met; or (3) high risk of bias if one or more criteria were not met.

### 2.5. Data Analysis

The meta-analysis was performed separately (reduction in pocket depth (PD) and gain in CAL), to identify the weighted mean change (WMD) in the effect of aPDT as an adjuvant to SRP, compared to SRP alone in smoking patients with periodontitis. The treatment effects were performed at 3 months and 6 months.

For the heterogeneity among the included studies, the I^2^ formula was taken as a measure, with values of I^2^ = 25%, I^2^ = 50%, and I^2^ = 70% indicating low, moderate, and high heterogeneity, respectively.

In case the heterogeneity was statistically significant (*p* < 0.05), the random effects model was employed, and the fixed effect model was employed if the heterogeneity was not significant. The alpha level was maintained at *p* ≤ 0.05 to determine statistically significant differences. Forest plots were produced to illustrate the effects in the meta-analysis, reporting WMD differences and 95% confidence intervals (95% CI). For the statistical analyses, the software RevMan (version 5.3.5, The Cochrane Collaboration, Copenhagen, Denmark) was used.

## 3. Results

### 3.1. Selection of Studies

**Figure 1:**
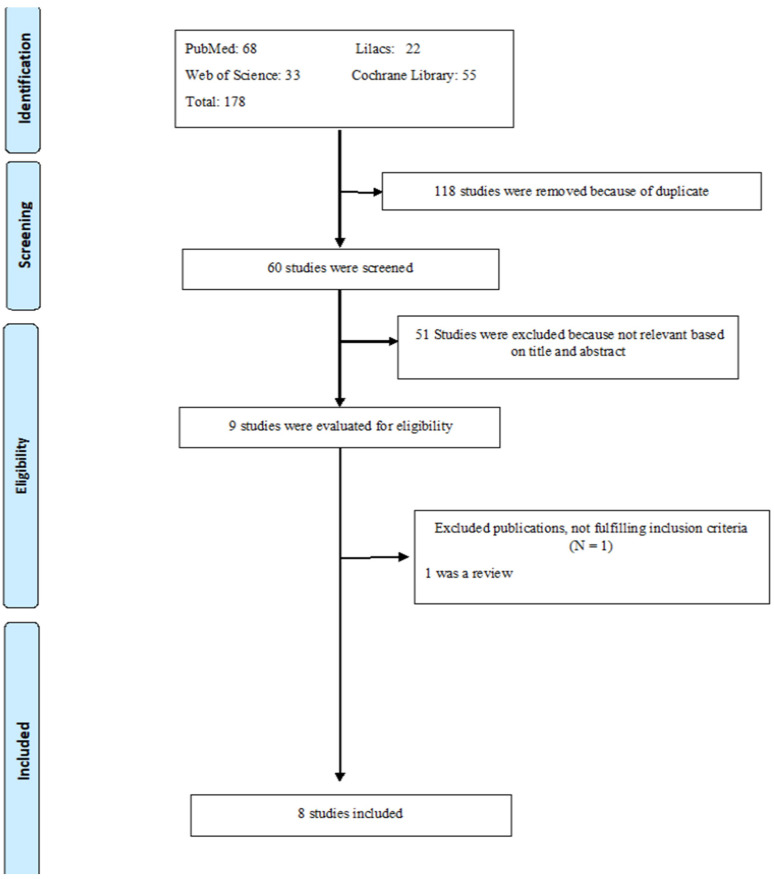
Flow chart of the selection strategy according to PRISMA guidelines.

Through the search strategy, a total of 178 studies were identified. After analysis, 118 were excluded for being duplicates. In total, 60 studies were selected; of these 51 were excluded after a review of the titles or abstracts. The full texts of the remaining nine publications were reviewed and, of these, eight studies [20,21,24,25,26,27,28,29] were included and one was excluded because it was a review article [30]. For a flowchart according to the PRISMA guidelines see Figure 1.

In this review, all the included studies were carried out at registered institutions, i.e., universities.

### 3.2. Characteristics of the Included Studies

The characteristics of the included studies are shown in Tables S1–S3. There were eight randomized clinical trials (RCTs) included, published between 2011 and 2022, with one study [24] having a patient assignment design and seven studies [20,21,25,26,27,28,29] having a split-mouth design. Of the eight studies, four were from Saudi Arabia [24,25,26,29] and the other four were from Brazil [20,21,27,28].

A total of 271 patients (208 men, 63 women) were initially enrolled, with 261 patients (202 men, 59 women) completing the follow-up period, resulting in a 95% completion rate. The average age for smoking patients ranged from 41.6 to 48 years, and the average age for the studies [24,25,26] that included non-smoking patients ranged from 40.5 to 46.9. In addition, seven studies [20,21,25,26,27,28,29] used the inclusion criteria of being a smoker and patients who smoked ≥10 cigarettes per day for 5 years or more, and only one study [24] indicated an average smoking history of 12.5 years.

### 3.3. Definition of the Disease

All the patients participating in the studies were diagnosed with periodontitis, six studies used the classification of generalized chronic periodontitis (CP) [20,21,24,25,26,29], and two studies [25,26] included a stage classification according to a 2017 workshop [31]. Six studies recruited patients with clinical attachment loss (CAL) ranging from ≥3mm [24,25,29] to ≥5 mm [20,21,26] and two studies did not mention the CAL value [27,28], but did mention the probing depth (PD). The values for the PD ranged from 4 to 6mm (PD 4mm [24], ≥5mm [20,21,25,27,28], and ≥ 6mm [26]), and one study [29] did not mention the PD value. Similarly, before starting periodontal therapy, two studies [20,21] classified PD as moderate pockets (values of 4–6mm) and deep pockets (≥7mm). The follow-up period for the clinical parameters varied from 1 month to 6 months.

### 3.4. Characteristics of Laser and Photosensitizer

The characteristics of the laser can be found in Table S2. Of the eight studies included in this review, six studies used a diode laser, one study used gallium-aluminium arsenide (GaAlAs) [20], and another study used aluminium gallium indium phosphide (InGaAlP) [25]. The wavelength ranged from 660 nm to 685nm, and seven studies [20,21,24,25,26,27,28] reported an energy fluency and output power that ranged from 2.5 J/cm^2^ to 160 J/cm^2^, and from 29 mW to 150 mW, respectively. Four studies [21,24,27,28] reported a radiation density between 28mW/cm^2^ and 75mW/cm^2^, and five studies [20,21,24,27,28] reported a fiber optic diameter that ranged from 0.03 to 0.6mm and the duration of irradiation ranged from 48 s to 60 s.

Regarding the photosensitizer used, three studies [21,27,28] used 100µg/mL chloro-phenothiazin, two studies [25,26] used chloro-aluminum phthalocyanine (CAP) with a concentration of 1.5 mg/mL, and three studies [20,24,29] used methylene blue with a concentration of 0.005% to 10 mg/mL.

### 3.5. Periodontal Therapy and Protocol for aPDT Administration

All the studies performed a full-mouth SRP under anesthesia, of which seven studies [20,21,24,25,27,28,29] used ultrasonic devices and manual instrumentation with Gracey (Hufriedy) curettes, and only one study performed scraping with manual curettes [24]. Seven studies [20,21,24,25,27,28,29] performed SRP in a single session, while one study [26] performed it in two sessions. After periodontal therapy, aPDT was performed. In five studies [20,21,24,27,28], the photosensitizer was placed in the periodontal pockets for a time that ranged from 10 s to 5 min, and in three other studies [25,26,29], the length of time that the photosensitizer was in the periodontal pocket was not mentioned. Regarding the number of PDT applications, De Melo Soarez et al. [21] used four aPDT applications, followed by Theodoro et al. [20] who used three applications, Al-Kheraif [25] who used two applications, and four studies [24,26,27,28] which used a single application throughout the study period. One study [29] did not mention the number of laser applications.

### 3.6. Clinical Parameter Results

The results of the clinical parameters are shown in Table S3. Seven studies [20,21,24,25,26,27,29] reported clinical parameter data (reduction of PD, gain of CAL). Of these, three studies [20,25,26] reported a follow-up period of 3–6 months, and four studies [21,24,28,29] reported a follow-up period of up to 3 months. The average reduction of PD varied from 3.28 mm [25] to 5.8 mm [24] in the aPDT group, and from 3.9 mm [29] to 5.5 mm [24] in the SRP group, at 3 months. The gain of CAL varied from 4.12 mm [20] to 9.24 mm [28] in the aPDT group, and from 4.51 mm [20] to 9.72 mm [28] in the SRP group, at 3 months. The average reduction of PD at 6 months varied from 2.97 mm [25] to 3.54 mm [20,26], while the gain of CAL varied from 4.11 mm [20] to 6.16 mm [26] in the aPDT group, and 4.47 mm [20] to 6.52 mm [26] in the SRP group.

### 3.7. Results of the Quantitative Assessment of PD and CAL

Seven studies were included in the quantitative evaluation, considering the effects of aPDT on changes in the periodontal clinical parameters (reduction of PD and gain of CAL). The overall effect for both was calculated using the weighted mean difference (WMD). The random effect model was used for the reduction of PD, as the heterogeneity was statistically significant at 3 and 6 months, respectively, (Chi^2^ = 32.37, *p* < 0.0001, I^2^ = 81% and Chi^2^ = 6.56, *p* = 0.04, I^2^ = 70%). Meanwhile, the fixed effect model was used for the gain of CAL, as there was no significant heterogeneity at 3 months (Chi^2^ = 5.24, *p* = 0.51, I^2^ = 0%).

**Figure 2:**
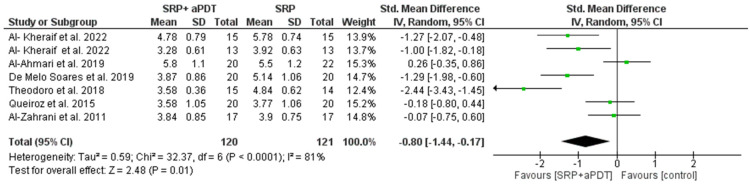
Forest plot of PD reduction at 3 months.

**Figure 3:**
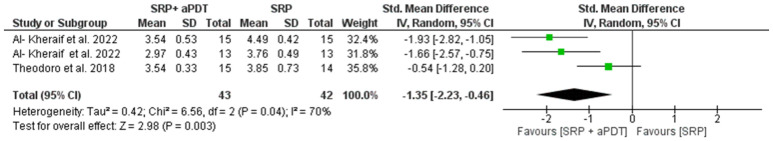
Forest plot of PD reduction at 6 months.

**Figure 4:**
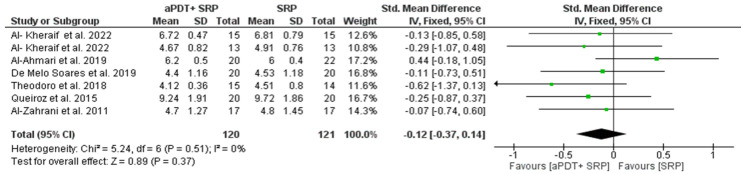
Forest plot of CAL gain at 3 month.

**Figure 5:**
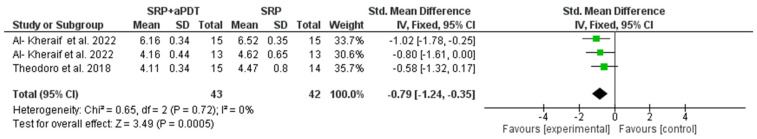
Forest plot of CAL gain at 6 months.

The overall effect for PD reduction at 3 and 6 months (WMD = −0.80, 95% CI = −1.44 to −0.17, *p* = 0.01, Figure 2) and (WMD = −1.35, 95% CI = −2.23 to −0.46, *p* = 0.003, Figure 3) was statistically significant in favor of the PDT group, while the CAL gain at 3 months (WMD= −0.12, 95% CI = −0.37 to 0.14, *p* = 0.37, Figure 4) was not statistically significant. Whereas gain in CAL at 6 months (WMD = 0.79, 95% CI = –1.24 to −0.35, *p* = 0.0005, Figure 5) was statistically significant in favor of aPDT.

### 3.8. Microbiological Parameters

#### 3.8.1. Sample Collection Site

Out of the eight included studies, only five studies performed microbiological analyses. Therefore, four studies [21,25,26,28] collected subgingival samples from the proximal tooth surface with a pocket depth (PPD) ≥ 5 mm, and one study [20] did not specify the tooth surface site but also indicated that the samples were collected from a PPD ≥ 5 mm. In addition, in the study by Theodoro et al. [20], subgingival plaque samples were collected according to the following PPD categories: moderate pockets (5 to 6 mm) and deep pockets (≥7 mm).

The collection time (follow-up) of the included studies ranged from 1 month [21,28] to 6 months [20,25,26].

#### 3.8.2. Microbiological Techniques Used

Out of the five studies that performed microbiological analyses, two studies [25,26] used the quantitative real-time polymerase chain reaction (RT-qPCR) technique to detect and quantify species associated with periodontitis such as *Porphyromonas gingivalis* (*Pg*) and *Tannerella forsythia* (*Tf*). Another study [20] used the StepOne PCR technique and the results were normalized against the 16S rRNA gene to detect the levels of *Pg*, *Prevotella intermedia* (*Pi*), and *Prevotella nigrescens* (*Pn*).

Meanwhile, two other studies [21,28] used the checkerboard DNA–DNA hybridization technique for 40 subgingival species. In these last two studies [21,28], the subgingival biofilm species were ordered into complexes according to Socranky et al. (1998) [32]. Some complexes were associated with periodontitis, such as the red complex (*Porphyromonas gingivalis* (*Pg*), *Treponema denticola* (*Td*), and *Tannerella forsythia* (*Tf*)), and the orange complex (*Campylobacter spp.*, *E. nodatum* (*En*), *Fusobacterium spp.*, *Prevotella spp.*, and *S. constellatus* (*Sc*)). The red complex was related to pocket depth and bleeding on probing [32].

#### 3.8.3. Microbiological Parameter Results

The results of the microbiological parameters are reported in Tables S4 and S5.

In the quantitative analysis, the total mean reduction (number of copies) of the *Pg* and *Tf* counts for the aPDT-treated smoking group varied from 3.457 [26] to 3.518 [25], and 8.464 [26] to 8.562 [25], respectively. For the SRP group, the total mean reduction (number of copies) of *Pg* and *Tf* counts varied between 961 [26]–971.47 [25], and 8.288.65 [26]–8.486 [25], respectively. Theodoro et al. [20] evaluated the levels (ng/mL) of *Pg*, *Prevotella intermedia* (*Pi*), and *Prevotella nigrescens* (*Pn*) in moderate and deep pockets at different time intervals (90 and 180 days). In moderate pockets, the aPDT group showed a higher amount of *Pg* than the SRP group at 180 days. Similarly, in deep pockets, there were higher levels of *Pg* in the aPDT group than in the SRP group at 90 days and 180 days. In moderate and deep pockets, *Pi* levels were higher in the aPDT group than in the SRP group at 180 days. While *Pn* levels were lower in the aPDT group than the SRP group at 90 and 180 days, both in moderate and deep pockets.

In the studies by De Melo Soarez et al. [21] and Queiroz et al. [28], through the checkerboard DNA–DNA technique, all 40 bacterial species were detected in different proportions and changes in the bacterial complexes were also observed in both groups at all follow-up periods. However, there was no significant difference between the groups.

In De Melo Soarez [21] et al., in the intra-group comparison, the control group showed a reduction in the red complex at 30 days compared to the baseline (*p* < 0.05), while the aPDT group showed a significant increase in the green complex at 90 days compared to the baseline.

A meta-analysis was not possible due to a limitation in the number of studies and the heterogeneity among the studies. In addition to using different microbial techniques, the results were presented in different units of measure. Therefore, the meta-analysis may be questionable.

#### 3.8.4. Secondary Results

The percentage changes in bleeding on probing (BOP) were reported in six clinical trials [20,21,24,25,26,29], which ranged from 16.59% [25] to 62.0% [21] in the PDT group, and from 24.66% [26] to 68.89% [20] in the SRP group. The plaque index was reported in five [21,24,25,26,29] studies, which ranged from 14.56% [26] to 41.90% [29], and from 17.83% [25] to 43.60% [29] in the PDT and SRP groups, respectively.

#### 3.8.5. Quality Assessment

The assessment of the methodological quality and the risk of bias in the studies was consistent between the two examiners (KRVV and CHM). According to the JADAD scale, four studies [20,21,25,26] had high methodological quality and four studies [24,27,28,29] had low methodological quality.

To evaluate the risk of bias, the Cochrane tool revealed that two studies [20,21,25,26] had a low risk of bias, two studies had an uncertain risk of bias, and four studies [24,27,28,29] had a high risk of bias. In the two studies [25,26] with an unclear risk of bias, the operator information was insufficient to judge the low or high risk of bias. Four studies [24,27,28,29] with a high risk of bias, inadequate allocation concealment, and insufficient masking methods (participants, operators, and evaluators) were insufficient for judgment. In all the clinical trials, the statistical analysis was adequate.

## 4. Discussion

This systematic review was based on RCTs and included eight studies. Of these, seven studies showed periodontal clinical results and five showed microbiological results.

There is concrete evidence that smoking is correlated with indicators of periodontal disease severity, including a greater number of lost teeth, a greater loss of attachment, and deeper probing [10]. Therefore, adjuvant treatments have emerged, including aPDT, which promotes microbial cell death through the combination of a photosensitizer, activated by a light source (laser), with the advantage of not generating microbial resistance [18,19]. Therefore, given the importance of the topic, this systematic review aimed to answer the question: What are the clinical and microbiological benefits of photodynamic antimicrobial therapy as an adjuvant to root scaling and planing in smokers with periodontitis?

The meta-analysis shows that adjuvant aPDT to SRP is more effective in reducing the PD and increasing the CAL than SRP alone. However, it is important to mention that of the seven studies, three studies [21,24,27] showed a reduction in PD and a greater increase in CAL in intragroup comparisons in favor of aPDT. However, these studies did not show statistically significant differences in the intergroup comparisons. Therefore, when observing the aPDT protocol in these studies, it was noted that two studies [21,27] used the same type of laser and a very similar laser configuration, and the diameter of the optical fiber was the same for the three studies [21,24,27]. The diameter of the optical fiber affects output power and energy fluence, which can affect the efficiency of photodynamic therapy [14].

Regarding the microbiological results, two studies showed a greater reduction in the counts of *Pg* [25,26], and *Tf* [25,26] species for the aPDT group compared to the SRP group. However, Theodoro et al. [20] showed higher levels of *Pg* and *Pi* at 90 and 180 days for the aPDT group compared to the SRP group. Meanwhile, only *Pn* species showed a greater reduction at 90 and 180 days in favor of the aPDT group compared to SRP in moderate and deep pockets. In addition, two other studies [21,28] did not show differences between groups. Smokers have a wide diversity of microorganisms in the biofilm, with a predominance of gram-negative bacteria such as *Pg*, *Tf*, and *Td*, known to contribute to the progression of the disease [33]. Therefore, aPDT has emerged as an adjuvant therapy to SRP with the aim of improving the efficiency of root scaling and reducing the number of pathogenic microorganisms. However, there is evidence that the absorption coefficient by bacteria depends on the specific photosensitizer and wavelength of the laser and these can have different effects on the periodontal tissues [34]. It was observed that the two studies [21,28] that did not show differences between groups used the same photosensitizer and the same wavelength (660 nm), as well as the same irradiation time of 60 s. However, one study [28] carried out a single application, and another [21] had multiple applications of aPDT. Systematic reviews [35,36] indicate that there were no statistically significant differences between single and multiple applications of aPDT to treat residual periodontal pockets [36] and to perform periodontal maintenance therapy [35]. Thus, it can be suggested that different results can be obtained from TPD, depending on the type of photosensitizer and the laser wavelength.

Regarding the photosensitizer used in the photodynamic therapy of the included RCTs, two studies [20,29] that used methylene blue associated with aPDT, with concentrations of 0.01% to 10mg/mL, showed better results compared to the phenothiazine chloride-based photosensitizer at 100 μg/mL. Some studies [37,38,39] suggest that photodynamic therapy associated with methylene blue may be effective in reducing the intensity of the local inflammatory response [38], promoting a better structural arrangement of the connective tissue [39], and reducing the depth of the periodontal pocket [37,38]. Two other studies [25,26] used chloro-aluminum phthalocyanine (CAP) at a concentration of 1.5 mg/mL and obtained statistically significant results in favor of the aPDT group for clinical and microbiological parameters compared to the SRP group. Some studies indicate that the CAP-assisted PDT mechanism as a photosensitizer improves fibroblast cell proliferation, with an increased deposition of the extracellular matrix [25,40]. Increased levels of fibroblasts allow for greater collagen formation, improving the gingival tissue integrity [40]. However, it is important to note that aPDT with CAP is an evolving area of research, and more studies are needed to evaluate its effectiveness.

Another important aspect is to research the effectiveness of photodynamic therapy in the immune response in smoking patients with periodontitis. This is because nicotine induces harmful effects on systemic health, reducing the activity of the immune system [41] and increasing the production of pro-inflammatory cytokine interleukins (IL)-1β [42] and tumor necrosis factor-alpha (TNF-α), thereby increasing the severity of periodontitis [43]. Of the studies included in this systematic review, four studies [21,25,26,27] evaluated the levels of the pro-inflammatory cytokines IL-1β [21,25], IL-6 [26], and TNF-α [21,25,26], the anti-inflammatory cytokine IL-10 [21,27], and the matrix metalloproteinase-8 (MMP8) [27], which is associated with the clinical manifestation of periodontitis [44]. In two studies [25,26], the levels of TNF-αwere higher in the aPDT group compared to the SRP group. In another study [27], although the effect of aPDT did not justify a greater improvement in clinical parameters in smokers, it resulted in the suppression of IL-1β [27] and MMP-8 [27] when compared to SRP alone. De Melo Soarez et al. [21] observed in moderate pockets that the levels of IL-10 were higher for the aPDT group, with a statistically significant difference compared to the SRP group on day 30. Statistical differences were observed within groups for IL-1β and IL-6 levels, although there were no intergroup differences. Therefore, the authors of the clinical trials included in this RS that evaluated immunological parameters indicate that aPDT-associated periodontal therapy helps reduce the inflammatory markers in smoking patients with periodontitis.

Reducing the inflammatory markers may also reduce the severity of periodontal disease, improving gingival health and periodontal support tissues (cementum, ligament, and alveolar bone), as well as decreasing BOP [45]. The studies [20,21,24,25,26,29] showed a reduction in BOP and PI after periodontal therapy; however, there were no differences in the intragroup and intergroup comparisons.

However, despite the results in favor of aPDT, it is important to mention some limitations of the studies. Half of the studies had low methodological quality, and the risk of bias was high in four studies and unclear in two studies, which could distort the results. In addition, most of the studies showed a short follow-up period, therefore more RCTs with longer follow-up periods are required. Furthermore, only studies published in English were considered, which could lead to the loss of other relevant studies in other languages. Therefore, the current findings are still limited and have to be interpreted with caution. Likewise, future studies are needed to establish standardized clinical protocols, with longer follow-up times, in order to provide more robust results on the effects of photodynamic therapy adjuvant to SRP in smokers with periodontitis.

## 5. Conclusions

Within the limitations of this SR it was observed that aPDT as an adjuvant to SRP in smoking patients with periodontitis more effectively improves clinical parameters (PD reduction and CAL gain). However, it remains debatable whether PDT is more effective in improving the microbiological parameters than SRP alone. Therefore, more RCTs with high methodological quality and longer follow-ups are needed to assess the efficiency of aPDT in smokers.

## Data Availability

Data sharing is not applicable to this article. All raw data supporting this systematic review are from previous literature studies, which have been cited in the text/figures.

## Supplementary Materials

The following supporting information can be downloaded at: https://www.mdpi.com/article/10.3390/medicina59040684/s1, Table S1: Characteristics of the studies; Table S2: Types and concentration of photosensitizers, time of photosensitizer, laser type, parameters and configurations of lasers (wavelength, energy fluence, power output, power density, optic fiber diameter), administration of aPDT (time and number of applications); Table S3: Type of periodontal disease and definition; interventions and follow-up; criteria for smoking/years smoking; periodontal parameters; main results in periodontal parameters; Table S4: Mean and standard deviation of the anaerobic microbiota of the subgingival plaque of the species selected by the included studies; Table S5: Microbial complexes (%) and mean counts (×10^5^) of 40 bacterial species. Comparing the treatment groups (SRP and SRP +aPDT) at follow-up; Table S6. Summary of assessment of quality and risk of bias (low +/high −/? unclear) in selected studies.
