# Supplementary material for: Benefits of Antimicrobial Photodynamic Therapy as an Adjunct to Non-Surgical Periodontal Treatment in Smokers with Periodontitis: A Systematic Review and Meta-Analysis
Source: Medicina (Kaunas). 2023 Mar 30;59(4):684. doi: 10.3390/medicina59040684 (PMC10142636; doi:10.3390/medicina59040684)
Supplement: Supplementary file 1 [file medicina-59-00684-s001.zip › Table_S1.pdf]

Table S1. Characteristics of the studies

Study and year of publication	Country	Study design	sample size (baseline)	Follow-up	Source of funding.
Al- Kheraif et al. 2022 [26]	Saudi Arabia	RCT<br>Split mouth	N=29 (23 male, 6 female)<br>14 Cigarette-smokers and 15 Non-smokers with Stage III Periodontitis<br>Mean age for cigarette smokers 42.2 ± 3.8 and Mean age for non-smokers 40.5±2.6	6 months	Research Supporting Project number (RSP- 2021/31), King Saud University, Riyadh, Saudi Arabia.
Al- Kheraif et al. 2022 [25]	Saudi Arabia	RCT<br>Split mouth	N=26 (23 male, 3 female)<br>13 Cigarette-smokers and 13 Non-smokers with Stage II Periodontitis<br>Mean age for cigarette smokers 45.2 ± 4.8 and Mean age for non-smokers 43.5 ± 3.6	6 months	Research Supporting Project number (RSP- 2021/31), King Saud University, Riyadh, Saudi Arabia.
AlAhmari et al. 2019 [24]	Saudi Arabia	RCT<br>Patients' assignment	N=83 (83 male)<br>42 Cigarette-smokers with CP and 41 Non-smokers with CP<br>Mean age for cigarette smokers 46.8 ± 3.2 and Mean age for non-smokers 46.9±2.82	3 months	Supported by International Scientific Partnership Program ISPP at King Saud University, Riyadh, Saudi Arabia ISPP#0075.
De Melo Soares et al. 2019 [21]	Brazil	RCT<br>Split mouth	N=22 (6 male, 16 female)<br>Age Range to 39- 61 years<br>Mean age of study 48 ± 6.4<br>only smoking patients	90 days	Funding The fiber tips and photosensitizers were donated by helbo (bredent medical GmbH & Co KG, Walldorf, Germany).
Theodoro et al. 2018 [20]	Brazil	RCT<br>Split mouth	N=51(35 male, 16 female)<br>Age range to 30 – 70 years<br>Mean age of study 48 ± 5<br>only smoking patients	180 days	Supported in part by FAPESP ("Support Foundation of São Paulo Research" Process # 2013/05424-3) and the CNPq (National Counsel of Technological and Scientific Development – Process 302806/2012-1) and ProInter/UNESP (Process # 2016-1346).
Queiroz et al. 2015 [27]	Brazil	RCT<br>Split mouth	N=20 (9 male, 11 female)<br>Age Range to 55 years<br>Mean age of study 46.05±6.38<br>only smoking patients	12 weeks	Supported by grants from the São Paulo State Research Foundation (grant2009/15845-0)
Queiroz et al. 2014 [28]	Brazil	RCT<br>Split mouth	N= 20(9 male, 11 female)<br>Age Range to 55 years<br>Mean age of study 46.05±6.38<br>only smoking patients	12 weeks	Supported by grants from the São Paulo State Research Foundation (grant2009/15845-0)
Al-Zahrani et al. 2011 [29]	Saudi Arabia	RCT<br>split-mouth	N=20 (20 male, 0 female)<br>Age Range to 30- 56 years<br>Mean age of the study 41.6 ± 9.6<br>only smoking patients	3 months	This study was supported by a grant from King Abdulaziz City for Science and Technology, Riyadh, Kingdom of Saudi Arabia (Grant No.LGP-14-32).
