# Supplementary material for: Benefits of Antimicrobial Photodynamic Therapy as an Adjunct to Non-Surgical Periodontal Treatment in Smokers with Periodontitis: A Systematic Review and Meta-Analysis
Source: Medicina (Kaunas). 2023 Mar 30;59(4):684. doi: 10.3390/medicina59040684 (PMC10142636; doi:10.3390/medicina59040684)
Supplement: Supplementary file 1 [file medicina-59-00684-s001.zip › Table_S2.pdf]

Table S2. Types and concentration of photosensitizers, time of photosensitizer, Laser type, parameters and configurations of laser (wavelength, energy fluence, power output, power density, optic fiber diameter), Administration of aPDT (time and the number of applications).

Study and year of publication	*Types and Concentration of photosensitizer*	*Time of photosensitizer*	*Laser type*	*Parameters and configurations of laser*					*Administration of aPDT (time/number of applications)*
				Laser Wavelength (nm)	*Energy Fluence (J/cm^2)*	*Power Output (mW)*	*Power density (mW/cm^2)*	*Optic Fiber Diameter (mm)*	
Al- Kheraif et al. 2022 [26]	Chloro- Aluminum Phthalocyanine (CAP) Topical application of CAP (25mg/ml) in conjunction with Gel-based delivery drug (1.5 mg/ml). This drug consisted of 23% (w/w) poloxamer 407 and Polyetilenoglicols (PEG 400) in a buffer solution pH -7.4	5 min	Diode laser (Thera Lase-DMC, São Carlos, Brazil)	685nm	3 J/cm^2	29mW	NR	NR	NR/ 1 application (immediately)
Al- Kheraif et al. 2022 [25]	Chloro- Aluminum Phthalocyanine (CAP) Topical application of CAP (30mg/ml) in conjunction with Gel-based delivery drug (1.5 mg/ml).<br><br>This drug consisted consisting of 23% (w/w) poloxamer 407 and Polyetilenoglicols (PEG 400) in a buffer solution (pH = 7.4)	5 min	InGaAlP (Thera Lase- DMC)	685 nm	2.5 J/cm^2	35mW	NR	NR	NR/ 2 application (immediately, 3 days)

AlAhmari et al. 2019 [24]	Methylene blue 0.005%	10s	Diode-Laser	660nm	NR	150mW	75mW/cm2	0.6 mm	60s/ application (immediately)
De Melo Soares et al. 2019 [21]	Phenothiazine Chloride 100 µg/ml (HELBO Blue Photosensitizer)	1 min	Diode-Laser (Helbo Photodynamic Sys., Walldorf, Germany)	660nm	16.72 J/cm2	70mW	28mW/cm2	0.6 mm	60s/ 4 applications (immediately, 2, 7, and 14 days)
Theodoro et al. 2018 [20]	Methylene blue 10 mg/ml (Aphoticário Manipulation Pharmacy, Araçatuba, SP, Brazil)	1min	GaAlAs (Laser Duo, MM Optics, Ltda, São Carlos, SP, Brazil)	660 nm	160 J/cm2	100mW	NA	0.03	48s/ 3 applications (immediately, 48h and 96h)
Queiroz et al. 2015 [27]	Phenothiazine Chloride 100 µg/ml (HELBO Blue Photosensitizer)	1min	Diode-Laser (Helbo Photodynamic Sys., Walldorf, Germany)	660 nm	16.72 J/cm2	60mW	28mW/cm2	0.6 mm	60s/ 1 application (immediately)
Queiroz et al. 2014 [28]	Phenothiazine Chloride 100 µg/ml (HELBO Blue Photosensitizer)	1min	Diode-Laser (HelboPhotodynami c Sys., Walldorf, Germany)	660 nm	16.72 J/cm2	60mW	28mW/cm2	0.6 mm	60s/ 1aplication (immediately)
Al-Zahrani et al. 2011 [29]	Methylene blue 0.01% (Ondine's Periowave, Ondine Biopharma Corp., Vancouver, BC).	NA	Diode-Laser	670nm	NR	NR	NR	NR	NR

NR, not reported; µg/ml, microgram/milliliter; mg/ml, milligram/milliliter; nm, nanometer; J/cm2, Joule/square centimeter; mW, milliwatts; mW/cm2, millwatt/square centimeter; s, seconds.
