# Supplementary material for: Benefits of Antimicrobial Photodynamic Therapy as an Adjunct to Non-Surgical Periodontal Treatment in Smokers with Periodontitis: A Systematic Review and Meta-Analysis
Source: Medicina (Kaunas). 2023 Mar 30;59(4):684. doi: 10.3390/medicina59040684 (PMC10142636; doi:10.3390/medicina59040684)
Supplement: Supplementary file 1 [file medicina-59-00684-s001.zip › Table_S3.pdf]

Table S3. Type of periodontal disease and definition; interventions and follow-up; criteria for smoking/ years smoking; periodontal parameters, main results in periodontal parameters

Study and year of publication	***Type of periodontitis and Definition***	***Interventions and Follow-up***	***criteria for smoking/ years of smoking***	***Periodontal parameters***			
				Probing depth (PD mm)	***Clinical Attachment level (CAL mm)***	***Bleeding on Probing (BOP%)***	***Plaque index (PI%)***
Al- Kheraif et al. 2022 [26]	Stage- III Generalized chronic periodontitis<br><br>PPD ≥ 6 mm, CAL≥ 5 mm, and MBL in contralateral teeth, tooth loss ≤4 teeth	**Group NS**<br>CAP/ aPDT+RSD<br><br>RSD alone<br><br>**Group CS**<br><br>CAP/aPDT+RSD<br><br>RSD alone<br><br>**Follow-up:**<br>Baseline,<br>3 months, and<br>6 months	Smokers ≥10 cigarettes per day for 5 years.	**Group NS**<br>**CAP/aPDT+RSD**<br>**Baseline**<br>6.12±0.23<br>**3 months**<br>4.67±0.21*†<br>**6 months**<br>2.94±0.89*<br><br>**RSD alone**<br>**Baseline**<br>6.21±0.71<br>**3 months**<br>4.41±0.53*†<br>**6 months**<br>3.43±0.41*<br><br>**Group CS**<br>**CAP/ aPDT+RSD**<br>**Baseline**<br>6.34±0.71<br>**3 months**<br>4.78±0.79*†<br>**6 months**<br>3.54±0.53*†<br><br>**RSD alone**<br>**Baseline**<br>6.43±0.51<br>**3 months**<br>5.78±0.74†<br>**6 months**<br>4.49±0.42*†	**Group NS**<br>**CAP/aPDT+RSD**<br>**Baseline**<br>5.22±1.14<br>**3 months**<br>4.18±0.88*†<br>**6 months**<br>4.03±0.49*<br><br>**RSD alone**<br>**Baseline**<br>5.56±1.03<br>**3 months**<br>4.72±0.95*†<br>**6 months**<br>4.59±0.74*<br><br>**Group CS**<br>**CAP/aPDT+RSD**<br>**Baseline**<br>7.45±1.25<br>**3 months**<br>6.72±0.47*<br>**6 months**<br>6.16±0.34*<br><br>**RSD alone**<br>**Baseline**<br>7.32±1.41<br>**3 months**<br>6.81±0.79*<br>**6 months**<br>6.52±0.35*	**Group NS**<br>**CAP/aPDT+RSD**<br>**Baseline**<br>54.23±18.24<br>**3 months**<br>19.52±4.70*<br>**6 months**<br>16.91±3.21*<br><br>**RSD alone**<br>**Baseline**<br>57.25±16.37<br>**3 months**<br>23.35±9.42*<br>**6 months**<br>20.89±8.31*<br><br>**Group CS**<br>**CAP/aPDT+RSD**<br>**Baseline**<br>40.32±7.94<br>**3 months**<br>27.45±6.25<br>**6 months**<br>18.71±5.32*<br><br>**RSD alone**<br>**Baseline**<br>35.41±6.82<br>**3 months**<br>24.19±7.92<br>**6 months**<br>25.45±7.21	**Group NS**<br>**CAP/aPDT+RSD**<br>**Baseline**<br>35.72±5.21<br>**3 months**<br>12.34±4.42*<br>**6 months**<br>10.45±2.21*<br><br>**RSD alone**<br>**Baseline**<br>39.32±9.21<br>**3 months**<br>14.25±3.12*<br>**6 months**<br>11.20±2.54*<br><br>**Group CS**<br>**CAP/aPDT+RSD**<br>**Baseline**<br>41.15±9.23<br>**3 months**<br>12.21±3.42*<br>**6 months**<br>14.56±2.37*<br><br>**RSD alone**<br>**Baseline**<br>42.14±10.12<br>**3 months**<br>16.36±6.12<br>**6 months**<br>19.41±7.23

Al- Kheraif et al. 2022 [25]	Stage- II Generalized chronic periodontitis<br><br>PD ≥ 5 mm, CAL of 3– 4 mm and horizontal alveolar bone loss in contralateral teeth	**Group NS**<br><br>CAP/PDT+DS<br>DS alone<br><br>**Group CS**<br>CAP/PDT+DS<br>DS alone<br><br>**Follow-up:**<br>Baseline,<br>3 months, and<br>6 months	Smokers ≥10 cigarettes per day for at least 5 years.	**Group NS**<br>**CAP/PDT+DS**<br>**Baseline**<br>4.64±0.55<br>**3 months**<br>3.12±0.28*†<br>**6 months**<br>2.89±0.21*<br><br>**DS alone**<br>**Baseline**<br>4.72±0.69<br>**3 months**<br>3.68±0.45*†<br>**6 months**<br>3.03±0.38*<br><br>**Group CS**<br>**CAP/PDT+DS**<br>**Baseline**<br>4.59±0.83<br>**3 months**<br>3.28±0.61*<br>**6 months**<br>2.97±0.43*†<br><br>**DS alone**<br>**Baseline**<br>4.51±0.77<br>**3 months**<br>3.92±0.63<br>**6 months**<br>3.76±0.49*†	**Group NS**<br>**CAP/PDT+DS**<br>**Baseline**<br>5.22±1.14<br>**3 months**<br>4.18±0.88*†<br>**6 months**<br>4.03±0.49*<br><br>**DS alone**<br>**Baseline**<br>5.56±1.03<br>**3 months**<br>4.72±0.95*†<br>**6 months**<br>4.59±0.74*<br><br>**Group CS**<br>**CAP/PDT+DS**<br>**Baseline**<br>5.83±1.25<br>**3 months**<br>4.67±0.82*<br>**6 months**<br>4.16±0.44*<br><br>**DS alone**<br>**Baseline**<br>5.46±1.41<br>**3 months**<br>4.91±0.76*<br>**6 months**<br>4.62±0.65*	**Group NS**<br>**CAP/PDT+DS**<br>**Baseline**<br>52.68±20.04<br>**3 months**<br>17.46±4.71*<br>**6 months**<br>14.87±2.90*<br><br>**DS alone**<br>**Baseline**<br>55.27±18.23<br>**3 months**<br>21.52±7.32*<br>**6 months**<br>18.69±6.51*<br><br>**Group CS**<br>**CAP/PDT+DS**<br>**Baseline**<br>38.71±6.82<br>**3 months**<br>25.42±5.81<br>**6 months**<br>16.59±4.93*<br><br>**DS alone**<br>**Baseline**<br>34.62±7.99<br>**3 months**<br>23.27±8.10<br>**6 months**<br>24.66±7.46	**Group NS**<br>**CAP/PDT+DS**<br>**Baseline**<br>34.61±8.63<br>**3 months**<br>11.82±3.46*<br>**6 months**<br>9.71±2.66*<br><br>**DS alone**<br>**Baseline**<br>38.45±10.34<br>**3 months**<br>13.82±4.01*<br>**6 months**<br>10.15±4.60*<br><br>**Group CS**<br>**CAP/PDT+DS**<br>**Baseline**<br>39.08±11.67<br>**3 months**<br>12.28±5.03*<br>**6 months**<br>14.72±6.66*<br><br>**DS alone**<br>**Baseline**<br>40.04±13.63<br>**3 months**<br>14.78±6.54*<br>**6 months**<br>17.83±8.09*
AlAhmari et al. 2019 [24]	Generalized chronic periodontitis<br><br>Presence of at least 30% of sites with CAL ≥ 3 mm and PD >3 mm	**Group 1 CS**<br>SRP alone<br>SRP+aPDT<br><br>**Group 2 NS**<br>SRP alone<br>SRP+aPDT<br><br>**Follow-up:**<br>Baseline,<br>1 month, and<br>3 months	Mean history of cigarette smoking of 12.5 years	**Group 1 CS**<br>**SRP alone**<br>**Baseline**<br>6.1 ± 0.5<br>**1 month**<br>5.4 ± 1.1<br>**3 months**<br>5.5 ± 1.2<br><br>**SRP+aPDT**<br>**Baseline**<br>6.4 ± 0.8<br>**1 month**<br>5.7 ± 0.4<br>**3 months**<br>5.8 ± 1.1	**Group 1 CS**<br>**SRP alone**<br>**Baseline**<br>7.1 ± 0.7<br>**1 month**<br>6.3 ± 0.6<br>**3 months**<br>6 ± 0.4<br><br>**SRP+aPDT**<br>**Baseline**<br>7.4 ± 0.7<br>**1 month**<br>6.4 ± 0.9<br>**3 months**<br>6.2 ± 0.5	**Group 1 CS**<br>**SRP alone**<br>**Baseline**<br>35.3 ± 6.9<br>**1 month**<br>30.6 ± 6.4<br>**3 months**<br>32.7± 2.4<br><br>**SRP+aPDT**<br>**Baseline**<br>31.6 ± 4.5<br>**1 month**<br>28.7 ± 2.8<br>**3 months**<br>28.2 ± 1.6	**Group 1 CS**<br>**SRP alone**<br>**Baseline**<br>52.6 ± 9.3<br>**1 month**<br>34.9 ± 8.4<br>**3 months**<br>38.5± 5.6<br><br>**SRP+aPDT**<br>**Baseline**<br>55.2 ± 5.6<br>**1 month**<br>30.3 ± 5.1<br>**3 months**<br>35.2 ± 3.9

				**Group 2 NS**<br>**SRP alone**<br>**Baseline**<br>6.6 ± 0.4<br>**1 month**<br>4.4 ± 0.8<br>**3 months**<br>4.1 ± 0.5<br>**SRP+aPDT**<br>**Baseline**<br>6.4 ± 0.5<br>**1 month**<br>4.6 ± 1.1<br>**3 months**<br>4.2 ± 0.8	**Group 2 NS**<br>**SRP alone**<br>**Baseline**<br>7.2 ± 0.5<br>**1 month**<br>5.2 ± 0.3<br>**3 months**<br>4.9 ± 0.6<br>**SRP+aPDT**<br>**Baseline**<br>7.1 ± 0.3<br>**1 month**<br>5 ± 0.6<br>**3 months**<br>5.2 ± 0.4	**Group 2 NS**<br>**SRP alone**<br>**Baseline**<br>61.2±9.6<br>**1 month**<br>17.3±4.5<br>**3 months**<br>20.3±4.7<br>**SRP+aPDT**<br>**Baseline**<br>65.4±6.6<br>**1 month**<br>19.4±8.1<br>**3 months**<br>21.6±5.4	**Group 2 NS**<br>**SRP alone**<br>**Baseline**<br>57.3±8.4<br>**1 month**<br>20.2±3.6<br>**3 months**<br>23.5±4.1<br>**SRP+aPDT**<br>**Baseline**<br>54.8±6.6<br>**1 month**<br>21.6±6.2<br>**3 months**<br>22.3±4.1
De Melo Soares M et al. 2019 [21]	Generalized Chronic periodontitis<br><br>Interproximal periodontal pockets with PD and CAL ≥ 5 mm in contralateral teeth	**Test Group**<br>SRP+aPDT<br><br>**Group Control**<br>SRP<br><br>**Follow-up:**<br>Baseline<br>30 and 90 days	Smokers ≥10 cigarettes per day at least 5 years	**Moderate pockets**<br>**SRP+aPDT**<br>**Baseline**<br>5.17±0.45<br>**30 days**<br>3.81 ± 0.85*<br>**90 days**<br>3.87±0.86*<br>**SRP**<br>**Baseline**<br>5.33±0.43<br>**30 days**<br>4.33 ±0.90*<br>**90 days**<br>4.13±1.09*<br><br>**Deep pockets**<br>**SRP+aPDT**<br>**Baseline**<br>7.29±0.95<br>**30 days**<br>6.64± 1.49<br>**90 days**<br>5.14±1.06*<br><br>**SRP**<br>**Baseline**<br>7.27±0.43<br>**30 days**<br>6.17± 1.62<br>**90 days**<br>5.50±1.11	**Moderate pockets**<br>**SRP+aPDT**<br>**Baseline**<br>5.25±0.59<br>**30 days**<br>4..41 ± 1.18*<br>**90 days**<br>4.40±1.16*<br>**SRP**<br>**Baseline**<br>5.34±0.95<br>**30 days**<br>4..47 ± 1.32<br>**90 days**<br>4.53±1.18<br><br>**Deep pockets**<br>**SRP+aPDT**<br>**Baseline**<br>7.59± 1.02<br>**30 days**<br>6.64± 1.13<br>**90 days**<br>5.87± 1.31*<br><br>**SRP**<br>**Baseline**<br>7.47±0.67<br>**30 days**<br>5.92±1.53*<br>**90 days**<br>5.70±1.46*	**SRP+aPDT**<br>**Baseline**<br>71.42<br>**30 days**<br>44.65<br>**90 days**<br>62<br><br>**SRP**<br>**Baseline**<br>65.45<br>**30 days**<br>31.18<br>**90 days**<br>53.06	**SRP+aPDT**<br>**Baseline**<br>67.85<br>**30 days**<br>33.92<br>**90 days**<br>34<br><br>**SRP**<br>**Baseline**<br>70.90<br>**30 days**<br>44.44<br>**90 days**<br>25

Theodoro et al.2018 [20]	Severe generalized chronic periodontitis<br><br>In at least 6 teeth, including one or several sites with PD ≥ 5 mm; CAL ≥ 5 mm; a minimum of 30% of the sites with PD and CAL ≥4 mm and BOP	**Group 1**<br>SPR<br>**Group 2**<br>SPR + MTZ-AMX<br>**Group 3**<br>SRP + aPDT<br><br>**Follow-up:**<br>Baseline, 90 and 180 days	Smokers ≥10 cigarettes per day for ≥5 years	**Group 1**<br>**SRP**<br>**Baseline**<br>4.19±0.85<br>**90 days**<br>4.84±0.62<br>**180 days**<br>3.85±0.73<br><br>**Group 3**<br>**SRP+aPDT**<br>**Baseline**<br>4.02±0.42<br>**90 days**<br>3.58±0.36*<br>**180 days**<br>3.54±0.33*	**Group 1**<br>**SRP**<br>**Baseline**<br>4.68±0.94<br>**90 days**<br>4.51±0.80<br>**180 days**<br>4.47±0.80†<br><br>**Group 3**<br>**SRP+aPDT**<br>**Baseline**<br>4.55±0.39<br>**90 days**<br>4.12±0.36*<br>**180 days**<br>4.11±0.34*†	**Group 1**<br>**SRP**<br>**Baseline**<br>76.48 ± 22.68<br>**90 days**<br>69.12 ± 19.44<br>**180 days**<br>68.89 ± 22.02<br><br>**Group 3**<br>**SRP+aPDT**<br>**Baseline**<br>87.88 ± 9.61<br>**90 days**<br>69.40 ± 13.08*<br>**180 days**<br>59.69 ± 17.97*	**NA**
Queiroz et al. 2015[27]	Chronic periodontitis<br><br>Presenting bilaterally at least 2 sites with PD ≥5 mm; at least 20 teeth present	**Group Test**<br>SRP+aPDT<br><br>**Group Control**<br>SRP<br><br>**Follow-up:**<br>Baseline, 4 weeks and 12 weeks	Smokers ≥10 cigarettes per day for ≥5 years	**SRP+aPDT**<br>**Baseline**<br>5.39 ± 0.74<br>**4 Weeks**<br>3.52 ± 0.82*<br>**12 weeks**<br>3.58 ± 1.05*<br><br>**SRP**<br>**Baseline**<br>5.35±0.70<br>**4 Weeks**<br>3.37 ± 0.98*<br>**12 weeks**<br>3.77 ± 1.06*	**SRP+aPDT**<br>**Baseline**<br>10.83 ± 1.45<br>**4 Weeks**<br>9.01 ± 1.70*<br>**12 weeks**<br>9.24 ± 1.91*<br><br>**SRP**<br>**Baseline**<br>11.12 ± 1.52<br>**4 Weeks**<br>9.28 ± 1.60*<br>**12 weeks**<br>9.72 ± 1.86*	**NA**	**NA**
Queiroz et al. 2014 [28]	Chronic periodontitis<br><br>Presenting bilaterally at least 2 sites with PD≥ 5 mm; at least 20 teeth	**Group Test**<br>SRP+aPDT<br>**Group Control**<br>SRP	Smokers ≥10 cigarettes per day for ≥5 years	**NA**	**NA**	**NA**	**NA**

Al-Zahrani et al. 2011 [29]	Generalized chronic periodontitis.<br><br>Marginal radiographic alveolar bone loss of >20% and CAL≥ 3 mm at 30% or more of sites.	**Group Test**<br>SRP+aPDT<br><br>**Group Control**<br>SRP<br><br>**Follow-up:**<br>Baseline and 3 months	Smokers ≥10 cigarettes per day for at ≥5 years.	**SRP+aPDT**<br>**Baseline**<br>5.60±0.83<br>**3 months**<br>3.84±0.85*†<br><br>**SRP**<br>**Baseline**<br>5.35±0.46<br>**3 months**<br>3.90±0.75*†	**SRP+aPDT**<br>**Baseline**<br>6.30±1.44<br>**3 months**<br>4.70±1.27*†<br><br>**SRP**<br>**Baseline**<br>6.18±1.40<br>**3 months**<br>4.80±1.45*†	**SRP+aPDT**<br>**Baseline**<br>74.50±21.50<br>**3 months**<br>41.90±22.30*<br><br>**SRP**<br>**Baseline**<br>68.00±23.00<br>**3 months**<br>45.60±19.50*	**SRP+aPDT**<br>**Baseline**<br>78.50±16.10<br>**3 months**<br>41.90±17.90<br><br>**SRP**<br>**Baseline**<br>75.90±15.80<br>**3 months**<br>43.60±16.60

PD, probing depth; CAL, clinical attachment level; NS, never smokers; CS, cigarette smokers; aPDT, antimicrobial photodynamic therapy; RSD, root surface debridement; DS, dental scaling; SRP, scaling root planing; *Intra-group, difference from baseline, $p < 0.05$; †Inter-group difference, significant differences between groups across same timeline ($p < .05$); NA, not evaluated.
