# Supplementary material for: Benefits of Antimicrobial Photodynamic Therapy as an Adjunct to Non-Surgical Periodontal Treatment in Smokers with Periodontitis: A Systematic Review and Meta-Analysis
Source: Medicina (Kaunas). 2023 Mar 30;59(4):684. doi: 10.3390/medicina59040684 (PMC10142636; doi:10.3390/medicina59040684)
Supplement: Supplementary file 1 [file medicina-59-00684-s001.zip › Table_S4.pdf]

Table S4. Mean and standard deviation of the anaerobic microbiota of the subgingival plaque, of the species selected by the included studies.

Study	Groups	*Porphyromonas gingivalis (Pg)*	*Tannerella forsythia (Tf)*	*Prevotella intermedia (Pi)*	*Prevotella nigrescens (Pn)*
		Mean± SD of copy number	Mean± SD of copy number	Mean± SD of copy number	Mean± SD of copy number
Al- Kheraif et al. 2022 [26]	**Group NS**<br>**PDT+ SRP**<br>Baseline<br>Follow up<br>3 months<br>6 months<br>Difference<br>3 months<br>6 months<br>**SRP**<br>Baseline<br>Follow up<br>3 months<br>6 months<br>Difference<br>3 months<br>6 months<br>**Group CS**<br>**PDT+ SRP**<br>Baseline<br>Follow up<br>3 months<br>6 months<br>Difference<br>3 months<br>6 months<br>**SRP**<br>Baseline<br>Follow up<br>3 months<br>6 months<br>Difference<br>3 months<br>6 months	**Group NS**<br>**aPDT+ SRP**<br>1.980.08±111.52‡<br>334.66±27.89*<br>1.366.53±111.56<br><br>**1.645.42**<br>613.55<br><br>**SRP**<br>1.590.7± 111.63‡<br>530.23±55.80*<br>2.065.12±139.53<br><br>**1.060.5**<br>-474.3<br><br>**Group CS**<br>**aPDT+ SRP**<br>6.203.36±332.17‡<br>575.67±57.92*<br>2.746.29±139.75*†<br><br>**5.627.35**<br>**3.457**<br><br>**SRP**<br>5.060.81±1.030.66‡<br>776.82±178.13*<br>4.099.96±827.04†<br><br>**4.283.18**<br>961	**Group NS**<br>**aPDT+ SRP**<br>1.932.29±130‡<br>281.25±46.87*<br>687.5± 67.7*<br><br>**1.651.04**<br>**1.245.29**<br><br>**SRP**<br>2.234.47 ± 139.53‡<br>747.93±54.07*<br>918.39±85.3*<br><br>**1.487.0**<br>**1.316.1**<br><br>**Group CS**<br>**aPDT+ SRP**<br>9.510.42 ±984‡<br>328.13±78*†<br>1.046.88±130.2*<br><br>**9.182**<br>**8.464**<br><br>**SRP**<br>10.235.7±1.025‡<br>938.60±128*†<br>1.946.35±212.38*<br><br>**9.296.4**<br>**8.288.65**		
Al- Kheraif et al., 2022 [25]	**Group NS**<br>**PDT+ DS**<br>Baseline<br>**Follow up**<br>3 months<br>6 months<br>**Difference**<br>3 months<br>6 months<br><br>**DS**<br>Baseline<br>Follow up<br>3 months<br>6 months<br>Difference<br>3 months	**Group NS**<br>**PDT+ DS**<br>1.821.47 ± 85‡<br>**160.81 ± 19.22***<br>1.202.78 ± 78.03‡<br><br>**1.661.97**<br>618.69<br><br>**DS**<br>1.504.61 ± 97.74‡<br>457.84 ± 29.62*<br>2.010.39± 124.32‡<br><br>**1.046.77**<br>-505.78	**Group NS**<br>**PDT+ DS**<br>1.760.61 ± 114.92‡<br>**108.79± 67.89***<br>540.69 ± 57.44<br><br>**1.651.82**<br>1.219.92<br><br>**DS**<br>1.963.9±164.65‡<br><br>453.68±111.9*<br>692.27± 92.67‡<br><br>**1509.32**<br>1.270.73	NA	NA

	6 months<br>**Group CS**<br>**PDT+ DS**<br>Baseline<br>Follow up<br>3 months<br>6 months<br>Difference<br>3 months<br>6 months<br>**DS**<br>Baseline<br>Follow up<br>3 months<br>6 months<br>Difference<br>3 months<br>6 months	**Group CS**<br>**PDT+ DS**<br>6.015.63 ± 300.37‡<br>**314.35 ± 33.29***†<br>2.497.26±142.23‡<br><br>**5.701.28**<br>3.518.37<br><br>**DS**<br>4.999.35±1.021.35‡<br>**713.97±145.06***†<br>4.027.87±796.35‡<br><br>**4.285.38**<br>971.48	**Group CS**<br>**PDT+ DS**<br>9.459.68 ± 940‡<br>**141.48± 83.56***<br>**897.14 ± 104.58**†<br><br>**9.408.2**<br>**8.562**<br><br>**DS**<br>10.257.2±1.072.1‡<br>748.31±142.41*<br>1.771.57±182.3‡†<br><br>**9.509**<br>**8.486**		
		Mean± SD (ng/mL)		Mean± SD (ng/mL)	Mean± SD (ng/mL)
Theodoro et al.2018 [20]	**Group 1**<br>**SRP**<br>Baseline<br>Follow up<br>90 days<br>180 days<br>Difference<br>90 days<br>180 days<br>**Group 3**<br>**SRP + aPDT**<br>Baseline<br>Follow up<br>90 days<br>180 days<br>Difference<br>90 days<br>180 days<br>**Group 1**<br>**SRP**<br>Baseline<br>Follow up<br>90 days<br>180 days<br>Difference<br>90 days<br>180 days<br>**Group 3**<br>**SRP + aPDT**<br>Baseline<br>Follow up<br>90 days<br>180 days<br>Difference<br>90 days<br>180 days	**Group 1**<br>**Moderate pockets**<br>**SRP**<br>1.21 ± 2.13<br>4.97 ± 17.92<br>0.45±1.68<br><br>-3.76<br>0.76<br>**Group 3**<br>**SRP + aPDT**<br>1.13 ± 2.40<br>0.97 ± 1.74<br>1.20 ± 2.13<br><br>0.16<br>-0.07<br><br>**Deep pockets**<br>**SRP**<br>0.88 ± 1.35<br>0.45 ± 0.79<br>1.08 ± 2.36<br><br>0.43<br>-0.2<br><br>**SRP + aPDT**<br>1.33 ± 5.15<br>2.51 ± 5.85<br>2.79 ± 6.20<br><br>-1.18<br>-1.46	NA	**Group 1**<br>**Moderate pockets**<br>**SRP**<br>0.31± 1.12<br>0<br>0<br><br>0.31<br>0.31<br>**Group 3**<br>**SRP + aPDT**<br>0.17 ± 0.39<br>0.02 ± 0.04<br>1.58 ± 5.77<br><br>0.15<br>-1.41<br><br>**Deep pockets**<br>**SRP**<br>0<br>0.02 ± 0.07<br>0.<br><br>-0.02<br>0.0<br><br>**SRP + aPDT**<br>**0**<br>0.01± 0.04<br>**0.07 ± 0.12***<br><br>0.01<br>-0.07	**Group 1**<br>**Moderate pockets**<br>**SRP**<br>0.33± 0.86<br>0.12 ± 0.37<br>0.02 ± 0.04<br><br>0.21<br>0.31<br>**Group 3**<br>**SRP + aPDT**<br>1.79± 6.89<br>0.01± 0<br>**0***†<br><br>1.78<br>1.79<br><br>**Deep pockets**<br>**SRP**<br>0.03± 0.07<br>0.07 ± 0.26<br>0<br><br>-0.04<br>0.03<br><br>**SRP + aPDT**<br>0.05 ± 0.12<br>**0***<br>**0***<br><br>0.05 ± 0.12<br>0.05 ± 0.12

NA, not evaluated; NR, not reported; *Intra-group, difference with baseline, $p < 0.05$; † Inter-group, indicates significant difference between test group (PDT+ SRP) and control group (SRP) across same timeline; ‡ Indicates significant difference between smokers and Never smokers.
