# Supplementary material for: Benefits of Antimicrobial Photodynamic Therapy as an Adjunct to Non-Surgical Periodontal Treatment in Smokers with Periodontitis: A Systematic Review and Meta-Analysis
Source: Medicina (Kaunas). 2023 Mar 30;59(4):684. doi: 10.3390/medicina59040684 (PMC10142636; doi:10.3390/medicina59040684)
Supplement: Supplementary file 1 [file medicina-59-00684-s001.zip › Table_S5.pdf]

Table S5. Microbial complexes (%) and mean counts ($x10^5$) of 40 bacterial species. Comparing the treatments groups (SRP and SRP +aPDT) at follow-up.

**Study**	**Groups**	**Microbial complexes (%)**					
		**Red color**	**Orange color**	**Purple color**	**Yellow color**	**Blue color**	**Green color**
De Melo Soares et al. 2019 [21]	**SRP+aPDT**<br>Baseline	18%	**34%†**	15%	5%	10%	6%
	Follow up<br>0 day	13%	**43%†**	8%	6%	12%	7%
	30 days	5%	40%	16%	5%	12%	10%
	90 days	8%	37%	12%	5%	12%	**9%***
	**SRP**<br>Baseline	16%	**41%†**	12%	5%	10%	6%
	Follow up<br>0 day	8%	42%	16%	6%	10%	8%
	30 days	**4%***	34%	15%	6%	17%	9%
	90 days	9%	36%	15%	6%	12%	8%
		**Mean counts** ($x10^5$) of 40 bacterial species for microbial complexes					
Queiroz et al. 2014 [28]	**SRP+aPDT**<br>Baseline	**Red color**<br>**16.6**<br>*T. forsythia* 7.21<br>*P. gingivalis* 40<br>*T. denticola* 2.62	**Orange color**<br>**5.52**<br>*C. gracilis:* 1.90<br>*C. rectus:* 2.86<br>*C. showae:*3.80<br>*E. nodatum:* 4.76<br>*F. nucleatum:* 3.49<br>*F. nucleatum ss polymorphum:* 5.39<br>*F. nucleatum ss vicentii:* 20.63<br>*F. periodonticum: 5.39*<br>*P. micra: 5.71*<br>*P. intermedia: 4.76*	**Purple color**<br>**4.85**<br>*A odontolyticus: 3.66*<br>*V. parvula:6.4*	**Yellow color**<br>**2.88**<br>*S. gordonii:3.88*<br>*S. intermedius:2.29*<br>*S. mitis: 2.29*<br>*S. oralis:1.6*<br>*S. sanguinis: 4.34*	**Blue color**	**Green color**<br>**3.24**<br>*A.actinomycetemcomitans:2.05*<br>*C. gingivalis:1.37*<br>*C. ochracea: 4.34*<br>*C. sputigena: 2.05*<br>*E. corrodens: 6.4*

			P. nigrescens: 4.76<br>*S. constellatus: 2.86*				
	Follow up<br>1 week/<br>4 weeks/<br>12 weeks	**16.8**<br>**16.41**<br>**17.73**<br>*T. forsythia:* 7.78/ 7.78/ 11.4<br>*P. gingivalis* 40/40/40<br>*T. denticola:* 2.62 / 1.47/ 1.80	**5.02**<br>**5.19**<br>**4.37**<br>*C. gracilis:*<br>2.52/ 2.10/ 1.43<br>*C. rectus:*<br>2.54/ 1.66/ 1.12<br>*C. showae:*<br>4.09/ 4.08/ 3.11<br>*E. nodatum:*<br>4.99/ 9.71/ 3.89<br>*F. nucleatum:*<br>2.27/ 3.92/ 3.0<br>*F. nucleatum ss polymorphum:*<br>4.81/ 4.8/ 4.8<br>*F. nucleatum ss vicentii:*<br>15.46/ 16.33/ 15.24<br>*F. periodonticum:* 5.29/ 5.06/ 4.84<br>*P. micra:*<br>5.85/ 3.99/ 4.75<br>*P. intermedia:*<br>3.56/ 3.46/ 4.33<br>*P. nigrescens:*<br>5.65/ 5.67/ 3.37<br>*S. constellatus:*<br>3.22/ 1.58/ 2.62	**5**<br>**9.1**<br>**4.17**<br>*A odontolyticus*<br>*3.73/3.18/2.63*<br>*V. parvula*<br>*6.27/15.03/5.72*	**3.68**<br>**2.97**<br>**2.86**<br>*S. gordonii:*<br>*5.72/4.86/4.02*<br>*S. intermedius:*<br>*2.47/1.92/2.1*<br>*S. mitis:*<br>*3.58/2.49/2.70*<br>*S. oralis:*<br>*1.68/1.41/1.62*<br>*S. sanguinis:*<br>*4.93/4.17/3.84*		**3.05**<br>**3.59**<br>**2.50**<br>*A. actinomycetemcomitans*<br>*1.80/1.28/0.90*<br>*C. gingivalis:*<br>*1.46/1.13/0.60*<br>*C. ochracea:*<br>*3.89/ 4.44/ 4.23*<br>*C. sputigena;*<br>*2.05/ 1.72/1.50*<br>*E. corrodens:*<br>*6.05/ 9.41/ 5.29*
	SRP<br>Baseline	**17.48**<br><br>*T. forsythia 9.83*<br>*P. gingivalis* 40<br>*T. denticola* 2.62	**6.16**<br><br>*C. gracilis:* 2.85<br>*C. rectus: 2.53*<br>*C. showae: 4.12*<br>*E. nodatum: 4.76*<br>*F. nucleatum: 4.12*	**5.03**<br><br>*A odontolyticus: 4.11*<br>*V. parvula: 5.94*	**3.83**<br><br>*S. gordonii: 8.91*<br>*S. intermedius: 2.7*<br>*S. mitis: 2.51*<br>*S. oralis: 1.6*<br>*S. sanguinis: 3.42*		**3.20**<br><br>*A. actinomycetemcomitans: 2.28*<br>*C. gingivalis:1.6*<br>*C. ochracea: 4.34*<br>*C. sputigena: 2.06*<br>*E. corrodens: 5.71*

			F. nucleatum ss polymorphum:5.07<br>*F. nucleatum ss vicentii: 24.44*<br>*F. periodonticum: 9.20*<br>*P. micra: 5.07*<br>*P. intermedia: 4.45*<br>*P. nigrescens: 4.44*<br>*S. constellatus: 2.85*				
	Follow up 1 week/ 4 weeks/ 12 weeks	**14.77**<br>**14.36**<br>**14.61**<br><br>*T. forsythia: 2.68/2.24/1.92*<br>*P. gingivalis: 38.95/ 38.62/40*<br>*T. denticola: 2.68/ 2.24/ 1.92*	**3.81**<br>**4.42**<br>**3.53**<br><br>*C. gracilis: 2.50/ 2.50/ 2.18*<br>*C. rectus: 2.50/ 2.18/ 1.96*<br>*C. showae: 3.27/ 3.60/ 3.05*<br>*E. nodatum: 5.78/ 5.45/ 5.46*<br>*F. nucleatum: 3.32/ 3.79/ 2.72*<br>*F. nucleatum ss polymorphum: 5.51/ 9.05/ 5.78*	**8.16**<br>**7.23**<br>**7.02**<br><br>*A odontolyticus: 4.32/ 3.96/ 3.73*<br>*V. parvula: 12/ 10.5/ 10.31*	**23.28**<br>**16.43**<br>**18.36**<br><br>*S. gordonii: 9.27/ 4.91/ 4.91*<br>*S. intermedius: 2.73/ 2.35/ 2.56*<br>*S. mitis: 3.84/ 2.83/ 3.20*<br>*S. oralis: 2.35/ 1.85/ 2.06*<br>*S. sanguinis: 5.09/ 4.49/ 5.63*		**17.19**<br>**15.91**<br>**15.33**<br><br>*A. actinomycetemcomitans: 2.18 / 2.07/ 2.34*<br>*C. gingivalis:1.88/ 1.60/ 1.08*<br>*C. ochracea: 4.28/ 4.58/ 4.07*<br>*C. sputigena: 2.51/ 1.81/ 2.12*<br>*E. corrodens: 6.34/ 5.85/ 5.72*

*Intra-group difference with baseline, $p < 0.05$; †Inter-group, significant difference between groups in the same period of analysis, $p < 0.05$.
