# Supplementary material for: Benefits of Antimicrobial Photodynamic Therapy as an Adjunct to Non-Surgical Periodontal Treatment in Smokers with Periodontitis: A Systematic Review and Meta-Analysis
Source: Medicina (Kaunas). 2023 Mar 30;59(4):684. doi: 10.3390/medicina59040684 (PMC10142636; doi:10.3390/medicina59040684)
Supplement: Supplementary file 1 [file medicina-59-00684-s001.zip › Table_S6.pdf]

Table S6. Summary of assessment of quality and risk of bias (low +/high - /? unclear) in selected studies

Study	Random sequence generation	Allocation concealment	Blinding subject	Blinding operator	Blinding examiner	Attrition bias	Selective reporting	Sample size calculation	Overall risk of bias	JADAD score
Al- Kheraif et al. 2022 [26]	+	+	+	?	+	+	+	+	?	4
Al- Kheraif et al. 2022 [25]	+	+	+	?	+	+	+	+	?	4
AlAhmari et al. 2019 [24]	+	-	-	-	-	+	+	?	-	2
De Melo Soares et al. 2019 [21]	+	+	+	+	+	+	+	+	+	5
Theodoro et al. 2018 [20]	+	+	+	+	+	+	+	+	+	5
Queiroz et al. 2015 [27]	+	-	-	-	-	+	+	+	-	2
Queiroz et al. 2014 [28]	+	-	-	-	-	+	+	+	-	2
Al-Zahrani et al. 2020 [24]	+	-	?	?	?	+	+	+	-	2
